# Knowledge, attitudes, practices and barriers of medical research among undergraduate medical students in Jordan: a cross-sectional survey

**DOI:** 10.1186/s12909-023-05002-9

**Published:** 2024-01-04

**Authors:** Mohammad Abusamak, Shahd AlQato, Hala Hani Alrfooh, Ruba Altheeb, Lujain Bazbaz, Rand Suleiman, Amjad Almansi, Alyaman Karajeh, Asem Alkhalaileh, Rasmieh Al-Amer

**Affiliations:** 1 Faculty of Medicine, Department of Special Surgery, Al-Balqa Applied University, AlSalt, Jordan; 2 Faculty of Nursing, Yarmouk University, Irbid, Jordan; 3 Ophthalmology, Amman Eye Clinic, 5435, 11183 Amman, Jordan

**Keywords:** Medical research, Barriers, Attitudes, Students, Knowledge, Survey

## Abstract

**Background:**

Integrating medical students in research at an early stage of their program is a crucial step to enhance the ability of future physicians to employ critical thinking and problem-solving processes, which in turn improves patients’ health outcomes.

**Methods:**

A cross-sectional questionnaire-based survey was administered to medical school students at Al-Balqa Applied University. This study aims to analyze the attitudes, practices, and barriers faced by medical students in regard to engaging in medical research.

**Results:**

A total of 333 students participated in the study with a mean age of 21.2 ± 1.5 years. A total of 60.1% were females. Female students had significantly higher knowledge scores (µ = 3.97, SD ± 1.81, P = 0.009) than males (µ = 3.44, SD ± 1.69). As students progress to higher academic years of their clinical program, their knowledge in research significantly increases in comparison to their knowledge in basic medical years (P < 0.001). Student age and academic year significantly correlated with the knowledge scores; each additional year of study increased the knowledge score by 0.25 (β = 0.25, P < 0.001, R^2^ = 0.63). The percentage of students who correctly answered each question on the knowledge scale was 37.5% (SD ± 12.5%). The most reported barriers to research participation were insufficient training in medical research, lack of sufficient research opportunities, and lack of stimulation and support from faculty.

**Conclusions:**

Medical students demonstrated a positive attitude toward research starting from their second year of study, despite having limited knowledge on the topic. They identified barriers that could be utilized to promote greater involvement of students in research. The implications for clinical practice suggest that policymakers and educators should consider the outcomes of this study and implement improvements in medical education, specifically by encouraging the early participation of students in the research process.

**Supplementary Information:**

The online version contains supplementary material available at 10.1186/s12909-023-05002-9.

## Introduction

Medical research plays a vital role in shaping the professional identity of medical students. It is an ongoing process that encourages curiosity, develops critical thinking skills, and enhances clinical judgment. Moreover, it helps students understand healthcare concepts, empowering them to contribute to the progress of their field and strengthening their professional identity. Medical students’ identities evolve around professional inclusion and social exclusion, with professional inclusion relating to their roles as physicians and professorial exclusion based on their perception of their status in other disciplines [1, 2]. To develop a professional identity, medical schools should focus on research early in their careers, allowing students to develop crucial skills such as writing a study proposal, collecting data, analyzing data, writing a literature review, and disseminating findings. This not only provides the competencies for quality care but also enhances their confidence in evidence-based decision-making, improving overall healthcare quality. Needless to say, the medical research process improves students’ critical thinking and problem-solving skills. The skills of searching for a topic, methodology formation, time management, and teamwork all improve the students’ approach to patient care [3, 4].

In 2022, a study conducted in six Arab countries, including Jordan, found that students had poor levels of knowledge about research skills but a positive attitude toward research; however, students identified the lack of access to lab equipment as the primary barrier to conducting research [5]. Despite scoring the highest on the barrier score, medical students in Jordan had high levels of knowledge and the most positive attitude toward research compared to students in the other included countries [5]. In 2015, a study conducted in Jordan revealed that students’ participation in research was very limited, despite their favorable attitude toward research, and cited insufficient training in medical research, a lack of research opportunities, and insufficient stimulation and support from faculty members as the most reported barriers to research participation [6].

Jordanian students are admitted through the General Secondary Education Certificate Examination in Jordan (also known as Tawjihi) competition system, requiring a minimum score of 85% to be eligible to study medicine, and the acceptance rate is extremely low [7]. The selection process for a residency program after finishing medical school is even more competitive [8]. Moreover, based on a study conducted in 2022, approximately one-third of Jordanian medical students expressed an intention to pursue their residency programs abroad, with the United States ranking as the primary destination, closely followed by the United Kingdom [9]. It has been observed that possessing research experience and engaging in related activities significantly enhances the chances of international students being accepted into their desired residency programs in either of these countries [10].

The existing body of literature on medical research does not sufficiently examine the viewpoint of medical students regarding the practicality of conducting such research. To fill this gap, the current study aims to gather factual information on how medical students at Al-Balqa Applied University perceive the research process. The study specifically intends to investigate the level of research knowledge among medical students, taking into account their previous experiences in high school or their current academic environment. In summary, this study aims to analyze the attitudes, practices, and barriers faced by medical students in regard to engaging in medical research.

## Methods

### Sampling and data collection

To investigate the research questions, a cross-sectional survey was developed. Undergraduate medical students at Al-Balqa Applied University campus were recruited who were willing to participate. The exclusion criteria were first-year students who studied outside the medical school campus.

The sample size was calculated using the Raosoft online sample calculator with a 95% confidence interval, 5% margin of error, and a total sample population of 1700 students [11]. This resulted in a sample size of 314 students, which was determined to be sufficient. To be conservative, the researchers aimed to recruit 10% extra; hence, the final sample size was planned to be 346. A multistage random table technique was used to sample the participants. First, the names of all the second- to sixth-year medical students’ names and emails were obtained from the admissions department and categorized into weighted strata based on their academic year, counts per class, and gender. Following this, every third name from the lists of male and female students in each academic year was selected, and the questionnaire was sent to their official university email address. In addition, an online consent form was obtained from each participant before accessing the questionnaire. We emailed a total of 370 questionnaires and received 350, of which 17 were excluded due to incompleteness or a deviation of responding to the questionnaire, such as selecting the first option for all questions, including the reversed questions. However, only 333 valid submissions were analyzed.

### Questionnaire

The questionnaire used in this study was developed based on a review of relevant literature as well as input from faculty members and students. The first section collected pertinent demographic characteristics of the participants, such as age, gender, and GPA; in addition, it explores the intended role of higher medical training for students with ambitions to pursue studies overseas. The next section addressed the involvement of students in research and publications within educational institutions, including both secondary schools and universities. It consisted of five Yes/No type questions about the involvement of students in high school research projects, the number of publications if they have any, and whether they had any oral or poster presentations during high school or in medical school. The third section measured the attitudes of undergraduate medical students toward research participation. This section comprises seven questions derived from a previous research study, with the exception of two questions that were reversed. On a 5-point Likert scale ranging from strongly disagree (score 1) to strongly agree (score 5), the responses were scored [12]. The fourth section explored the barriers to taking part in research, adopted from a previous study [6], which consisted of eight Likert-type questions that discussed medical students’ opinions on training opportunities in research methods, available research facilities, and available medical staff working in research. The fifth part assessed the students’ knowledge of research, adopting questions from a previous study [13]. It consisted of 10 multiple-choice questions with four options. The questionnaires in this study demonstrated reliability, as evidenced by a Cronbach’s alpha coefficient of 0.7–0.8. The final version of the questionnaire was pilot tested on 30 medical students from the faculty and was modified accordingly; these students were not included in the study sample. A copy of the questionnaires and scales were included in Supplementary_file_1.

### Statistical methods

This study used SPSS software (version 28.0.0.1) to analyze and manage the data. We presented continuous variables using measures of central tendency, such as the mean and standard deviation, and categorical variables (e.g., gender) using frequency tables. The independent t test was used to compare study-related variables by gender (the outcome variables were attitude score, knowledge score, and research participation). One-way ANOVA was utilized to examine the relationship between dependent variables and academic year, intention to study abroad, and GPA. We conducted a post hoc test to determine the exact difference between groups.

Descriptive and analytic statistics were used to summarize demographic characteristics, barriers, and other dependent variables in the tables. The attitude score was calculated by assigning 1 to strongly agree and agree, 0 to neutral, and − 1 to disagree and strongly disagree. The students’ knowledge score was determined by the number of correct responses on a 10-question scale. A higher score indicates a higher level of knowledge, with scores ranging from 0 to 10. A *P*-value of 0.05 was considered statistically significant.

## Results

### Descriptive statistics and population characteristics

The mean age of the cohort (*n* = 333) was 21.2 years (SD = 1.5 years), with a range of 19 to 28 years. The preponderance of students (60.1%) was female. The majority of students were in their second or third year of medical school. Overall, 57.1% of students had a very good GPA score, while 11.7% had an excellent score. The majority of students intended to complete their residency abroad (59.8%), while 35.4% were undecided. The overwhelming majority of students (96.1%) had completed the Tawjihi national high school examination program, as demonstrated in Table 1.

**Table 1:** Characteristics of the studied students

Characteristics		n = 333
Age in years		21.2 ± 1.5 (19–28)
Gender		
Male, n (%)		133 (39.9)
Female, n (%)		200 (60.1)
Study Year		
Second, n (%)		85 (25.5)
Third, n (%)		73 (21.9)
Fourth, n (%)		87 (26.1)
Fifth, n (%)		47 (14.1)
Sixth, n (%)		41 (12.3)
GPA		
Excellent, n (%)		39 (11.7)
Very Good, n (%)		190 (57.1)
Good, n (%)		104 (31.2)
Intention to do residency abroad		
No, n (%)		16 (4.8)
Yes, n (%)		199 (59.8)
Not sure, n (%)		118 (35.4)
High school program		
Tawjihi, n (%)		320 (96.1)
IG, n (%)		9 (2.7)
IB, n (%)		1 (0.3)
SAT, n (%)		3 (0.9)

### Students’ attitudes toward research

The majority of medical students (79.7%) agreed or strongly agreed that research should be part of the Medical Doctor (MD) curriculum. Moreover, a significant proportion of students (81.3%) agreed or strongly agreed that research would help them better understand the subject. Additionally, 29.1% of students felt that research was an extra burden, while 88.5% agreed or strongly agreed that it would help their clinical practice later, as illustrated in Table 2. Finally, a majority of students (75.9%) disagreed or strongly disagreed that research was a waste of time and disturbed their studies. However, only 51.8% of students agreed or strongly agreed that their research record should be an important criterion for acceptance into residency as illustrated in Table 2.

**Table 2:** Percentages of medical students’ answers on the attitude scale

Attitude Items		Strongly Disagree	Disagree	Neutral	Agree	Strongly Agree
1	Research should be part of MBBS curriculum	1 (0.3)	5 (1.5)	59 (17.7)	135 (40.5)	133 (39.9)
2	Research will NOT help in better understanding of subject	112 (33.6)	159 (47.7)	45 (13.5)	14 (4.2)	3 (0.9)
3	It is an extra burden to do research	33 (9.9)	92 (27.6)	111 (33.3)	84 (25.2)	13 (3.9)
4	Research will help ones clinical practice later	1 (0.3)	6 (1.8)	31 (9.3)	158 (47.4)	137(41.1)
5	It is a waste of time and it disturbs studies	119 (35.7)	134 (40.2)	59 (17.7)	18 (5.4)	3 (0.9)
6	Your research record should be an important criterion for acceptance in residency	14 (4.2)	40 (12.0)	106 (31.8)	110 (33.0)	63 (18.9)

**Notes**: Data are presented as n and %

### Inferential statistics

Table 3 illustrates a comparison of average attitude and knowledge scores with respect to medical students’ gender, academic year, intention to perform residency abroad and GPA. The mean knowledge score was 3.76 (SD 1.78). Female students scored better on both the attitude and knowledge scales; however, the difference on the attitude scale was not significant (*P = 0.056*). The ANOVA test suggests that the knowledge score differ significantly between academic years (*F*_4,328_= 6.18, *P* < 0.001), with higher scores among 6th and 5th year medical students when compared to the second year. However, no significant differences were detected between both years or between 3rd and 4th years by post-hoc analysis using Bonferroni correction that was conducted after confirming that Levene’s test was not significant and Equal variance was assumed. Both the mean score of 6th year (*M* = 4.39, *SD* = 1.56) and 5th year (*M* = 4.36, *SD* = 1.84) were significantly different from the mean score of 2nd year (*M* = 3.07, *SD* = 1.62). In contrast, the difference in attitude scale was not significant between different academic years (*P = 0.418*). Students who intended to perform their residency abroad and those who are undecided had significantly more positive attitudes toward research than students who did not intend to perform their residency abroad (*P* = 0.021), nevertheless post-hoc did not detect a significant difference between these three groups. In addition, students with excellent GPAs had significantly more positive attitudes toward research than other students (*P* = 0.025) and got the highest average attitude score (*M* = 4.08, *SD* = 0.57) however, post-hoc detected a significant difference only between average attitude score of students with excellent GPAs and those with good GPAs.

A supplementary table displaying further information regarding the percentages of Al-Balqa Applied University respondents who answered correctly to questions in the knowledge section is provided in Supplementary_file_1.

**Table 3:** Comparison of knowledge and attitudes of medical students in relation to research by gender, academic year, intention to perform residency abroad and GPA.

		Knowledge		Attitude	
		Mean ± SD	*p* – value	Mean ± SD	*p* - value
**t test**					
**Gender**					
	Female	3.97 ± 1.81	0.009*	3.90 ± 0.50	0.056
	Male	3.44 ± 1.69		3.82 ± 0.59	
**ANOVA test**					
**Academic year**					
	Second	3.07 ± 1.62		3.81 ± 0.50	
	Third	3.77 ± 1.97		3.87 ± 0.48	
	Fourth	3.79 ± 1.63	< 0.001*	3.94 ± 0.56	0.418
	Fifth	4.36 ± 1.84		3.93 ± 0.63	
	Sixth	4.39 ± 1.56		3.80 ± 0.58	
**Intention to do residency abroad**					
	No	3.81 ± 1.80		3.67 ± 0.48	
	Yes	3.77 ± 1.88	0.974	3.94 ± 0.56	0.021*
	Not sure	3.73 ± 1.62		3.79 ± 0.50	
**GPA**					
	Excellent	3.77 ± 1.77		4.08 ± 0.57	
	Very good	3.91 ± 1.79	0.148	3.87 ± 0.51	0.025*
	Good	3.48 ± 1.75		3.81 ± 0.58	
**Total**		3.76 ± 1.78		3.87 ± 0.54	

**Note**: ‘*’ stars next to statistically significant values. **SD***stands for* standard deviation

In Table 4, a multivariate linear regression analysis was conducted to examine the factors predicting knowledge and attitude among students. Residency intention was not a significant predictor of either attitude or knowledge about research. Age, academic year, and the intention to pursue residency training abroad were not statistically significant factors in determining students’ attitudes. Our analysis shows that academic year and age clearly were strong predictors of knowledge, with positive regression coefficients of 0.250 (*P* < 0.001) and 0.253 (*P* < 0.001), respectively. This means that for every one-year increase in academic year or age, students’ knowledge about research increased by 0.25 units.

**Table 4:** Predictor of medical students’ knowledge and attitude scores

	R-squared		Regression coefficient (Beta)	*P*-value
**Attitude**				
Academic year		0.01	0.03	0.590
Age		0.007	0.082	0.133
Residency intention		0.004	-0.061	0.268
**Knowledge**				
Academic year		0.63	0.250	< 0.001*
Age		0.064	0.253	< 0.001*
Residency Intention		0.000	-0.013	0.817

**Note**: ‘*’ stars next to statistically significant values

### Barriers to participation in research

The most remarkable results to emerge from the data on barriers to conducting research were insufficient training in medical research (84.4%), lack of sufficient research opportunities (72.9%), lack of stimulation and support from faculty members (71.1%), lack of adequate research facilities and resources (68.4%), and lack of sufficient funding (68.4%), as outlined in Table 5.

**Table 5:** Students’ opinions on barriers to research

Barriers Items	Strongly Disagree	Disagree	Neutral	Agree	Strongly Agree
Insufficient training in medical research.	2 (0.6)	11 (3.3)	39 (11.7)	168 (50.5)	113 (33.9)
Lack of sufficient research opportunities.	1 (0.3)	14 (4.2)	77 (23.1)	148 (44.4)	93 (27.9)
Lack of stimulation and support from faculty members.	6 (1.8)	23 (6.9)	67 (20.1)	136 (40.8)	101 (30.3)
Lack of adequate research facilities and resources.	6 (1.8)	31 (9.3)	68 (20.4)	135 (40.5)	93 (27.9)
Lack of sufficient funding	7 (2.1)	21 (6.3)	77 (23.1)	134 (40.2)	94 (28.2)
Medical students do not have sufficient time	12 (3.6)	40 (12.0)	68 (20.4)	119 (35.7)	94 (28.2)
Faculty members do not have sufficient time.	9 (2.7)	40 (12.0)	115 (34.5)	112 (33.6)	57 (17.1)
Lack of sufficient faculty members	9 (2.7)	64 (19.2)	104 (31.2)	106 (31.8)	50 (15.0)

**Notes**: Data are presented as ***n*** and ***%***. Likert scale: 1 = Strongly disagree to 5 = Strongly agree

Table 6 demonstrates that there was no statistically significant difference in research practice by gender (*P* = 0.585). However, it is interesting to note that female medical students were more likely to have research experience in both high school and university (51.7%) than male students (48.3%). On the other hand, academic year was statistically significant in research practice by students (*P* < 0.001). Moreover, there was no statistically significant difference in research practice by intention to perform residency abroad (*P* = 0.133). However, students who intended to perform residency abroad were more likely to have research experience in both high school and university (72.4%) than students who did not intend to perform residency abroad (0.0%). Finally, there was no statistically significant difference in research practice by GPA (*P* = 0.439).

**Table 6:** Research practice by gender, age, intention to do residency abroad, and GPA.

Characteristics		Research Practice experience							*P-*value
		No	High School		University		Both		
**Gender**	Female, n (%)	85 (42.5)		47 (64.4)		53 (59.6)		15 (51.7)	0.585
	Male, n (%)	57 (40.1)		26 (35.6)		36 (40.4)		14 (48.3)	
**Academic Year**	Second, n (%)	41 (28.9)		34 (46.6)		6 (6.7)		4 (13.8)	
	Third, n (%)	26 (18.3)		18 (24.7)		19 (21.3)		10 (34.5)	
	Fourth, n (%)	52 (36.6)		20 (27.4)		10 (11.2)		5 (17.2)	< 0.001*
	Fifth, n (%)	13 (9.2)		1 (1.4)		29 (32.6)		4 (13.8)	
	Sixth, n (%)	10 (7)		0 (0)		25 (28.1)		6 (20.7)	
**Intention to do Residency Abroad**	No, n (%)	8 (5.6)		6 (8.2)		2 (2.2)		0 (0)	
	Yes, n (%)	81 (57)		40 (54.8)		57 (64)		21 (72.4)	0.133
	Not sure, n (%)	53 (37.3)		27 (37)		30 (33.7)		8 (27.6)	
**GPA**	Excellent, n (%)	15 (10.6)		5 (6.8)		15 (16.9)		4 (13.8)	
	Very Good, n (%)	77 (54.2)		49 (67.1)		50 (56.2)		14 (48.3)	0.439
	Good, n (%)	50 (35.2)		19 (26)		24 (27)		11 (37.9)	

## Discussion

The most remarkable findings of this study suggest that female students who are seniors and have higher GPAs possess greater knowledge and more positive attitudes toward research. This may be due to several factors, including having had greater exposure to research opportunities, receiving more mentorship from faculty members, and being more motivated to pursue careers in research. It is also interesting to note that students who intended to complete their residency abroad or who were undecided had higher attitude scores than those who were not interested in studying abroad, according to our analysis. This is partially explicable by the fact that research experience is not a prerequisite for residency training in Jordan, whereas research aptitude and experience are highly valued in Western countries. An alternative rationale could be that students who are enthusiastic about undertaking their residency overseas are more inclined to perceive research as an essential element of their medical education and career development. In general, the results of this study indicate that a number of variables may impact the understanding and perspectives of medical students regarding research. Understanding these factors is necessary in order to formulate strategies that foster positive attitudes toward research and motivate students to participate in research activities.

The findings of the present research revealed that the mean total knowledge score was 37.6%. It is important to note that a higher score is indicative of greater levels of knowledge. The aforementioned findings provide corroborating evidence from prior research conducted in India and Iran [12, 14]. Furthermore, the present study has shown a notable disparity in knowledge across genders, wherein females exhibit superior scores of knowledge compared to males. The findings of this study align with prior research conducted in six Arab nations, which indicated that females exhibit greater dedication toward educational objectives [5, 13]. One plausible hypothesis posits that the cultural norms prevalent in the Arab world tend to value women who successfully attain admission to prestigious educational institutions, such as medical schools.

The knowledge scores of medical students demonstrated a positive correlation with their progression through the academic years. The participants in their sixth year demonstrated the greatest total knowledge score (4.39), while the second-year students exhibited the lowest score (3.07), as demonstrated in Table 3. It is expected that senior students will be needed to fulfill their school’s graduation requirements by submitting a full research project. Research conducted in Pakistan showed comparable results, as it revealed a steady increase in knowledge with the completion of each consecutive academic year according to **Khan et al., (2006)**. The current study findings align with previous research conducted in six Arab nations that similarly reported that students in the clinical phase of their training (i.e., the years leading up to graduation) had superior knowledge compared to their counterparts in the foundational phase of their education [5]. The plausibility of these findings stems from the observation that when students advance toward completing their academic program, they undergo a significant shift in their development, marked by the emergence of their professional identity. Nevertheless, research conducted in India revealed that the students in their second year exhibited the highest level of knowledge, with the final-year students ranking second in terms of information acquisition. The study conducted by **Pallamparthy & Basavareddy (2019)** suggests that the observed phenomenon may be attributed to methodological concerns, specifically pertaining to the sample methodologies employed.

Remarkably, our analysis revealed a lack of statistical significance when examining the relationship between academic year and students’ attitudes toward research. These findings are incongruous with previous studies that have reported a positive correlation between attitude and academic progression, as observed in research undertaken in Saudi Arabia, Pakistan, the United States, and India [12, 13, 15, 16]. The cited studies have explored various aspects of the topic under investigation. This finding may be attributed to the difficulties faced by students in understanding the research process, as a limited number of medical students in Jordan have been successful in disseminating their research findings throughout publications, posters, etc.

Moreover, the results of the study indicate that there is no discernible correlation between gender and research attitude **(**Table 3**)**. While it was previously noted that Arabic culture holds a favorable view of women pursuing medical studies, it is important to consider the quantifiability of knowledge. In this regard, female students have demonstrated higher levels of knowledge compared to their male counterparts. However, when examining attitudes, no significant differences based on gender were found. This may be attributed to the subjective nature of attitudes, making it challenging for peers to accurately assess them. Similar findings were observed in other studies undertaken in India, Saudi Arabia, Iran, and Croatia, which also examined the academic performance of first-year medical students [12, 15, 17, 18]. Two studies conducted in Pakistan investigating undergraduate and postgraduate doctors revealed that males exhibited a more favorable attitude toward research than their female counterparts [13, 19].

Furthermore, the present study identified several barriers to learning and participation in research, as illustrated in Table 5. The most comparable findings to our study were obtained from an earlier study conducted in 2015, which focused on undergraduate students from Jordan [6]. The current study reported that the primary barrier was a lack of adequate training in medical research, with a percentage of 82.4%, which is in line with the findings of, who highlighted the inadequate training of students in research methodologies as a potential barrier [6].

Another major barrier we have identified among Al-Balqa Applied University students is a lack of sufficient time due to their academic workload, which affects 28.2%. This barrier has been observed in numerous prior studies and has been reported at significantly higher rates [5, 6]. The limited availability of time among medical students can be considered a valid barrier due to the demanding nature of their curriculum and the extensive hours needed for clinical training. Furthermore, the barrier that was least frequently identified by participants was the insufficiency of faculty members, with only 15% of respondents indicating this as a hindrance. The findings of research conducted on students from Jordan were consistent with those cited earlier [6]. In contrast, a study conducted in Saudi Arabia revealed that a significant barrier mentioned by students was the absence of supervisors, with a prevalence rate of 73.3% [15].

The progress of students’ practice exhibited improvement throughout the academic year, with the exception of fourth-year students, who demonstrated comparatively lower levels of participation in comparison to their counterparts in previous years. This is explained by the fact that students begin their clinical training in the fourth year, which is intensive and demanding. The present finding aligns with previous research conducted in Saudi Arabia, India, and Yarmouk University in Jordan [12, 15, 20, 21], where comparable findings were revealed, indicating a positive association between seniority and improved research practice. This finding is consistent with the results of a research study undertaken in six Arab nations, including Jordan, which revealed a positive correlation between an advanced academic year and improved research practices [5]. The aforementioned discovery diverged from a study conducted among undergraduate students in Saudi Arabia (specifically Riyadh) and India, where no notable disparity was observed among medical students based on their level of academic year [12, 22].

The present survey observed no statistically significant difference in practice between male and female students. Additional research conducted at Yarmouk University, Saudi Arabia (Riyadh), India, and six Arab countries, including Jordan, has yielded similar findings, indicating no discernible gender differences [5, 12, 21, 22]. In contrast, a study conducted in Saudi Arabia found that females had better practice scores [15]. There was no statistically significant difference in research participation among the medical students involved in the study, according to their GPAs. The present finding bears a resemblance to a study carried out at Yarmouk University in Jordan [21].

### Implications

The findings of this study have several implications for medical education and research. First, it is important to provide all medical students with equal opportunities to learn about research and to participate in research activities. This may be done by integrating research into the medical curriculum, providing mentorship programs for students, and offering financial support for research projects. Second, it is important to address the barriers that may prevent students from engaging in research. These barriers may include a lack of time, a lack of resources, and a lack of confidence in their research skills. Finally, it is important to recognize the importance of research in medical education and career development. This can be done by promoting research among students and by rewarding faculty members for their research contributions. Future research could examine the longitudinal relationship between knowledge and attitude toward research and other factors, such as students’ prior experiences with research, their career aspirations, and their research engagement. Future research could also be conducted at multiple institutions to increase the generalizability of the findings.

### Limitations

This study has some limitations owing to its cross-sectional design, as it is unable to demonstrate a causal relationship or evaluate the research question over time. Other factors, such as students’ prior research experiences or career aspirations, may have contributed to the observed differences in knowledge and attitudes toward research. The study was conducted at a single medical school, which is a significant limitation. The findings may not be generalizable to medical students attending other medical schools.

## Conclusions

The present study investigated the knowledge, attitudes, and barriers toward research among medical students at Al-Balqa Applied University in Jordan. The findings suggest that female students, students in their later years of study, and students with higher GPAs have better knowledge and more positive attitudes toward research. A lack of sufficient time due to their academic workload was the second most frequently cited barrier by students after a lack of adequate training in medical research. The identified barriers can be minimized to promote student research participation. This may be done by integrating research into the medical curriculum, providing mentorship programs for students, and offering financial support. Noting that our study was conducted at a single university, we recommend that future research be expanded to include the remaining six universities in Jordan to increase generalizability.

### Electronic supplementary material

Below is the link to the electronic supplementary material.

Supplementary_file_1. Excel spread sheet contains two tabs: Supplementary Table S1 that includes supplementary table S1 displaying further information regarding the percentages of Al-Balqa Applied University who answered correctly to questions in the knowledge section. Supplementary data includes all the study’s questionnaires.

## Data Availability

The datasets used and/or analyzed during the current study are available from the corresponding author upon reasonable request.
